# Microbial diversity in the hypersaline Lake Meyghan, Iran

**DOI:** 10.1038/s41598-017-11585-3

**Published:** 2017-09-14

**Authors:** Ali Naghoni, Giti Emtiazi, Mohammad Ali Amoozegar, Mariana Silvia Cretoiu, Lucas J. Stal, Zahra Etemadifar, Seyed Abolhassan Shahzadeh Fazeli, Henk Bolhuis

**Affiliations:** 1 Department of Biology, Faculty of Science, University of Isfahan, Isfahan, Iran; 2 Microorganisms Bank, Iranian Biological Resource Centre (IBRC), ACECR Tehran-Iran, Tehran, Iran; 3 Extremophiles Laboratory, Department of Microbiology, Faculty of Biology and Center of Excellence in Phylogeny of Living Organisms, College of Science, University of Tehran, Tehran, Iran; 4 Department of Marine Microbiology and Biogeochemistry (MMB), NIOZ Royal Netherlands Institute for Sea Research and Utrecht University, Texel, The Netherlands; 5 Department of Freshwater and Marine Ecology, IBED, University of Amsterdam, Amsterdam, The Netherlands; 6 Department of Molecular and Cellular Biology, Faculty of Basic Sciences and Advanced Technologies in Biology, University of Science and Culture, Tehran, Iran

## Abstract

Lake Meyghan is one of the largest and commercially most important salt lakes in Iran. Despite its inland location and high altitude, Lake Meyghan has a thalassohaline salt composition suggesting a marine origin. Inputs of fresh water by rivers and rainfall formed various basins characterized by different salinities. We analyzed the microbial community composition of three basins by isolation and culturing of microorganisms and by analysis of the metagenome. The basins that were investigated comprised a green ~50 g kg^−1^ salinity brine, a red ~180 g kg^−1^ salinity brine and a white ~300 g kg^−1^ salinity brine. Using different growth media, 57 strains of Bacteria and 48 strains of Archaea were isolated. Two bacterial isolates represent potential novel species with less than 96% 16S rRNA gene sequence identity to known species. Abundant isolates were also well represented in the metagenome. Bacteria dominated the low salinity brine, with Alteromonadales (Gammaproteobacteria) as a particularly important taxon, whereas the high salinity brines were dominated by haloarchaea. Although the brines of Lake Meyghan differ in geochemical composition, their ecosystem function appears largely conserved amongst each other while being driven by different microbial communities.

## Introduction

Hypersaline ecosystems are widely distributed habitats including a variety of terrestrial lakes and deep-sea basins with salt concentrations exceeding three times seawater up to saturation^1^. In addition to being hypersaline, these ecosystems are often characterized by other extremes in environmental conditions such as high alkalinity, low oxygen concentration and high UV irradiation^2–4^. Hypersaline habitats can be divided into two main types, thalassohaline- and athalassohaline waters^5^. Thalassohaline waters or brines are of marine origin and have an ionic composition similar to that of seawater, with sodium chloride as the predominant salt and are often found in close proximity to seas and oceans. These include industrial solar salterns and natural shallow basins that became detached from the sea or ocean^3, 6^. Athalassohaline waters or brines such as the Dead Sea and soda lakes are often found inland and therefore not directly connected to marine waters. These brines are shaped by the dissolution of mineral salt deposits of continental origin, which are dominated by potassium-, magnesium-, sodium- and carbonate ions^7^. Although high salinity is generally considered lethal for most organisms, hypersaline environments are often teeming with life and can harbor high biomass of functional and taxonomical diverse communities^8–10^.

Iran has a large diversity of hypersaline habitats, only a few of which have been investigated with respect to their microbial community composition. Analysis of the culturable microbial diversity in the Aran-Bidgol salt lake revealed isolates that belong to the genera *Halorubrum*, *Haloarcula*, *Salinibacter*, *Salicola*, and *Rhodovibrio* ^11^ while from Lake Urmia mainly bacteria were isolated belonging to the Proteobacteria (21.4%), Firmicutes (78.6%), and Actinobacteria (1.8%)^12^. Further exploration of the Iranian salt lakes is of interest for potential biotechnological applications such as the biological treatment of saline wastewater and the production of - for example - β-carotene, compatible solutes, bioplastics, salt tolerant enzymes, and biofuels^13, 14^.

Lake Meyghan covers an area of 100–110 km^2^ in the central part of Iran (Markazi province, north of Arak city) and is surrounded by mountains with heights between 2,000–3,000 m above sea level. The distances to the nearest seashores are 450 km to the Persian Gulf and 350 km to the Caspian Sea. The latter is characterized by a low salinity of 12 g kg^−1^ and, hence, is an intermediate between a sea and a lake. The Caspian Sea has been formed by a tectonic depression that drains an extensive catchment area^15^. High evaporation and a lack of rainfall in recent years in this region has led to an increased salinity^15^. The lake itself is located at an altitude of 1,660 m in an area with an arid to semi–arid continental climate and the temperature ranges from −30 °C in winter to 40 °C in summer. The lake receives average annual precipitation of ~320 mm and the maximum lake depth during the wet season is ~1.5 m, while the average annual evaporation is ~2,070 mm. Ephemeral rivers along with smaller rivulets deliver sediment-loaded rainwaters from the catchment area into the lake basin. Lake Meyghan is commercially one of the most important hypersaline lakes in Iran because of its mineable sodium sulfate deposits, which is the largest in the Middle East^15^. Despite its origin and location, which could have resulted in an athalassohaline composition like the divalent cation-rich Dead Sea^16^, Lake Meyghan is sodium chloride-dominated with a high sulfate concentration of 48–62 g L^−1^ ^15^, more than three times the concentration of Great Salt Lake (10–20 g L^−1^)^17, 18^ and a slightly higher pH (7.7–8.8) than other salt lakes. Besides the presence of birds that feed on the abundantly present brine shrimp - *Artemia salina* - nothing is known about the biodiversity in the hypersaline parts of the lake. Here we describe the first metagenomic analysis of the microbial community composition in an Iranian salt lake. In addition, we performed a cultivation dependent analysis and reveal that the microbiota of Lake Meyghan share commonalities with both thalassohaline and athalassohaline hypersaline lakes and that each of the stations exhibit their own microbial signature.

## Results

### Site description

**Figure 1:**
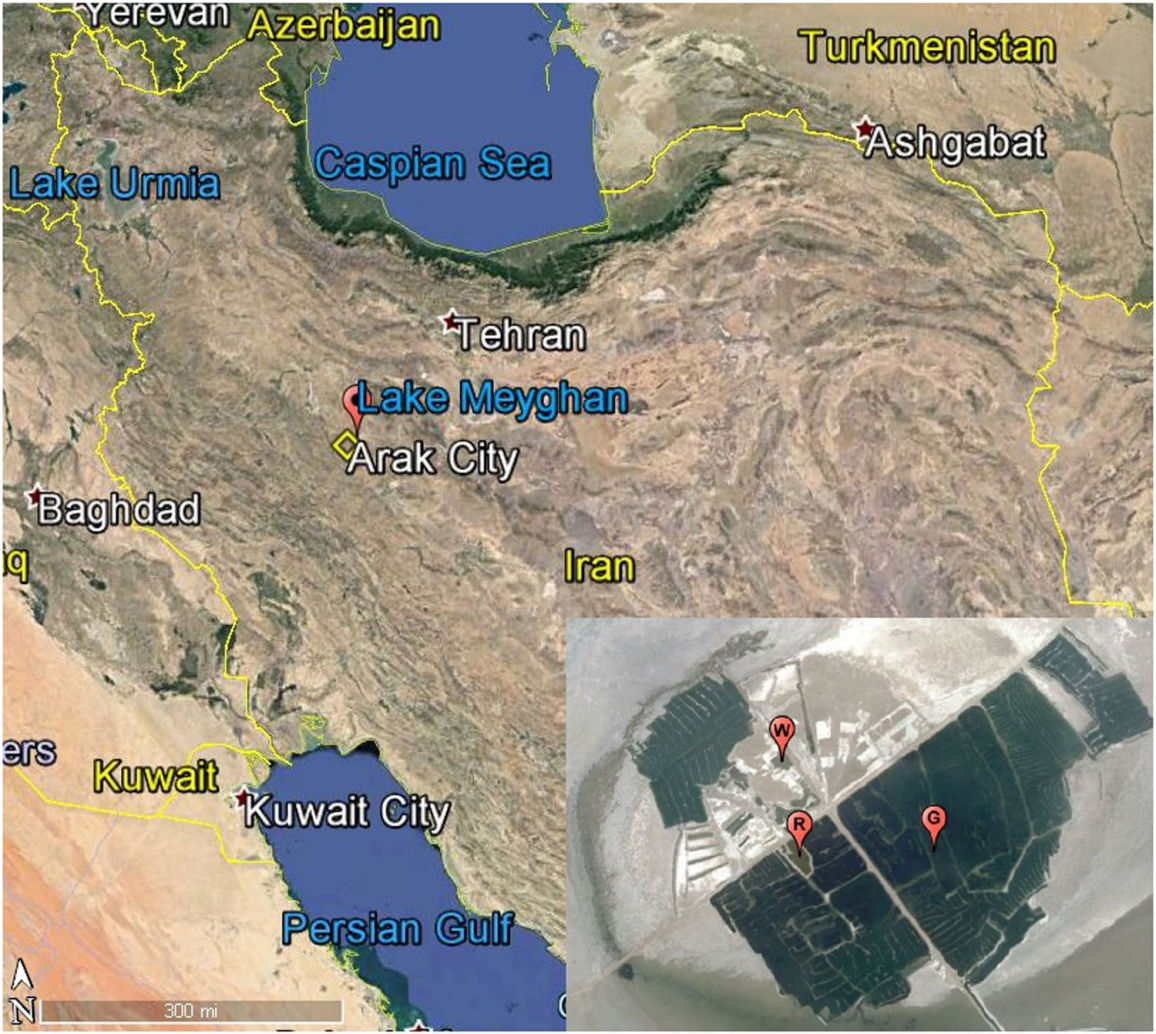
Geographic location of Lake Meyghan (see red marker left) and sampling sites denoted in the inset right with the letters G, R, and W. Google Earth Pro version 7.1.5.1557, © 2015 Google Inc.

The shallow brines of Lake Meyghan were sampled at three different sites that were named according to the dominant brine color, i.e. G (green), R (red) and W (white) (Fig. 1). The physicochemical properties of the brine samples are presented in Table 1. Na^+^ and Cl^−^ were identified as the major ions in the three samples, followed by SO_4_^2−^ and Mg^2+^. With a pH of 8.8 (G), 7.9 (R), and 7.7 (W), the brines were moderately alkaline with salinities of 50 g kg^−1^, 180 g kg^−1^, and 300 g kg^−1^, respectively. The bacterial and archaeal abundance was estimated by qPCR using domain specific 16S rRNA primers (Table 2). The sum of the archaeal and bacterial 16S rRNA gene copies was used to estimate the total prokaryote abundance. Total 16S rRNA gene copy number decreased by ~50% with increasing salinity from 8.1 × 10^6^ (G) to 3.6 × 10^6^ (W) copies per ml. The relative contributions of Bacteria and Archaea also changed with increasing salinity. Bacteria were the dominant group (79%) at low salinity (G) and Archaea were dominant (84%) at the highest salinity (W).

**Table 1:** Physicochemical properties of the water from the 3 sample sites in Lake Meyghan.

Site	S (g kg^−1^)	pH	T (°C)	Ion concentration (g L^−1^)								
				Ca^2+^	Mg^2+^	Fe^2+^	Na^+^	K^+^	Mn^+^	Cl^−^	SO_4_^2−^	HCO_3_^−^
G	50	8.8	15	0.38	0.4	<0.0001	8.6	0.06	<0.0001	11.6	4.9	0.167
R	180	7.9	15	0.56	2.5	0.0002	64.7	0.22	<0.0001	92.6	22.2	0.766
W	300	7.7	15	0.55	2.5	<0.0001	111.5	0.15	0.0001	164.1	21.2	0.225

**Table 2:** Total prokaryote, bacterial and archaeal 16S rRNA gene copy abundance.

Site	Total prokaryote 16S rRNA genes (10^6^ copies mL^−1^)	Number and % of 16S rRNA gene copies per domain			
		Bacteria	%	Archaea	%
G	8.1	6.4 × 10^6^	79	1.7 × 10^6^	21
R	5.5	1.2 × 10^6^	22	4.3 × 10^6^	78
W	3.6	5.6 × 10^5^	16	3.0 × 10^6^	84

### Bacterial and archaeal isolates

A total of 361 strains was isolated using different media. From each site, the 16S rRNA gene of 35 randomly chosen isolates (105 in total) was sequenced (Supplementary Table S1, sheet 9). The low salinity pond (G) yielded mainly Bacteria (31 out of 35). Proteobacteria were the dominant group with 20 Gammaproteobacteria (e.g. *Idiomarina* sp. and *Halomonas* sp.) and 3 Alphaproteobacteria. The other bacterial isolates belong to the phyla of Actinobacteria (3), Bacteroidetes (4), and Firmicutes (1). From the medium-salinity red pond (R), 16 Bacteria were isolated of which 9 belong to the Firmicutes (e.g. *Bacillus* sp. and *Thalassobacillus*), 5 Gammaproteobacteria, and 2 Actinobacteria. Bacteroidetes and Alphaproteobacteria were not amongst the isolates from the red pond. From the highest salinity, 10 Bacteria were retrieved, 6 of which belong to the Gammaproteobacteria, 3 Firmicutes, and 1 Alphaproteobacteria. Of the sequenced isolates 4, 19, and 25 were of archaeal origin from the Green (G), Red (R), and White (W) sample sites, respectively (Supplementary Table S1, sheet 9). All archaeal isolates belong to three families within the class of Halobacteria: Halobacteriaceae, Haloferacaceae and Natrialbaceae with respectively *Haloarcula*, *Halorubrum*, and *Natrinema* as the dominant genera. We did not obtain Haloferacaceae in the collection of the lower salinity brine isolates while they dominate the collection of isolates at the highest salinity (site W, 10 isolates).

### Metagenome community analysis

**Figure 2:**
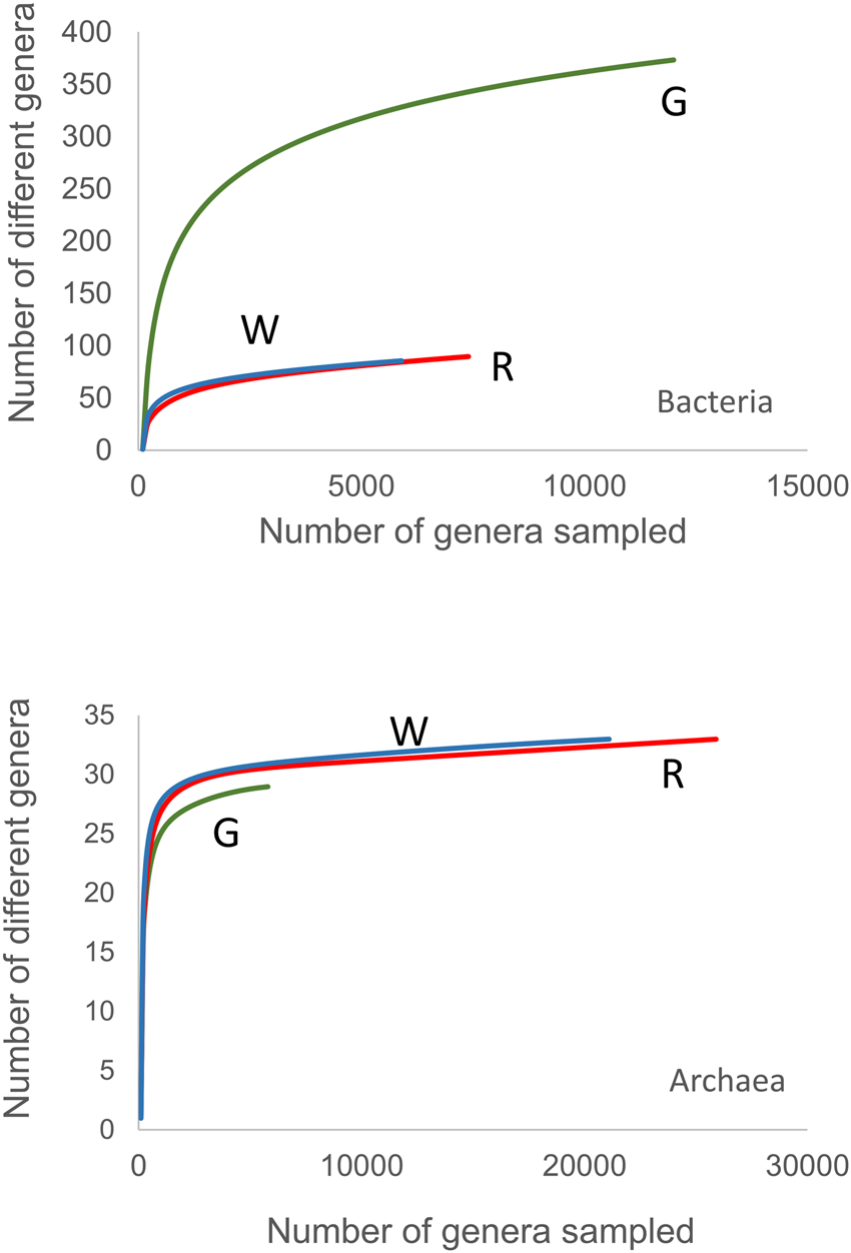
Rarefaction curves obtained from MG-RAST annotated rRNA genes, clustered at the genus level for Bacteria (top-panel) and Archaea (bottom panel).

**Figure 3:**
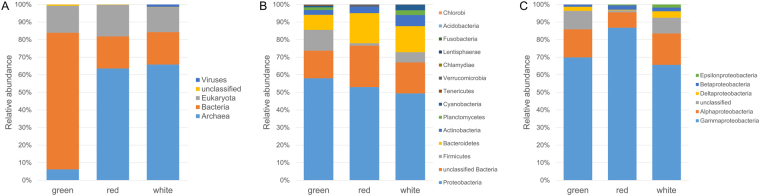
Relative distribution of domains (**A**), bacterial phyla (**B**) and proteobacterial classes (**C**) at the three sampling sites as deduced from the metagenomic dataset.

The metagenome of the Meyghan lake communities yielded per site ~35 million paired sequence reads of ~100 nucleotides each (Table 3). MG-RAST identified 6.8 million (G), 4.1 million (R), and 3.0 million (W) reads, corresponding with alignment-identified protein features in green, red, and white samples, respectively. Due to the insecurity in correctly assigning the relatively small sequencing reads at the species level we further analyzed the taxonomic annotation derived from rRNA fraction in MG-RAST at the genus level (Supplementary Table S1 sheet 10). Rarefaction curves obtained from the genus assigned rRNA fraction of the metagenomes for the bacterial and archaeal populations are nearing an asymptote showing that the sequencing depth is sufficient to identify the majority of abundant genera but may miss a number of rare genera (Fig. 2). The green site is dominated by bacteria (78%) (Fig. 3A) with an estimated richness of 374 genera while the hypersaline lakes have an estimated richness of 90 (R) and 86 (W) bacterial genera. Archaea dominate the hypersaline lakes (64%, red and 66% white) (Fig. 3A), but with an estimated richness of 29 (G) and 33 (W + R), the archaeal richness is much lower than that of Bacteria. Bacterial genus diversity using the Shannon diversity index was estimated at 5.1 (G), 2.8 (R) and 3.3 (W). The archaeal diversity was estimated at 2.5 (G), 1.8 (R) and 2.4 (W).

**Table 3:** Sequence read statistics.

Parameter	Green	Red	White
Base pair count	3,677,922,376	3,523,663,474	3,691,100,649
Total sequence count	36,670,335	35,135,376	36,823,996
Sequences passing QC	31,229,554	29,432,222	28,796,277
Mean sequence length	100 ± 4 bp	100 ± 4 bp	100 ± 3 bp
GC percentage	54 ± 12%	61 ± 9%	61 ± 8%
Alignment identified protein features	6,849,136	4,127,650	3,026,598
Alignment identified rRNA features	18,708	6,263	4,474
Alignment identified functional categories	5,437,468	2,940,129	2,180,056

In all brines we found approximately 15% of the reads belonging to Eukarya. The dominant bacterial phyla were, in order of abundance, Proteobacteria, Firmicutes, Bacteroidetes, and Actinobacteria, which made up more than 94% of the total bacterial diversity in the three brines (Fig. 3B). In addition, a large number of bacterial reads was found that could not be assigned to any known phylum. In the hypersaline brines (W and R), Bacteroidetes were more abundant than Firmicutes. Notably, two phyla, Planctomycetes and Cyanobacteria, were present at the lowest as well as at the highest salinity (albeit at low abundance) but were absent from the intermediate salinity brine (R). The dominant proteobacterial class in all samples were Gammaproteobacteria (Fig. 3C) with, in order of abundance, Alteromonadales, Vibrionales, Pseudomonadales, Oceanospirillales, and Enterobacteriales as dominant orders at low salinity (G) while at medium high salinity (R) Oceanospirillales, Pseudomonadales, and Alteromonadales dominated and at the highest salinity (W) Enterobacteriales, Pseudomonadales, and Legionellales dominated (Supplementary Table S1, sheet 4). Alphaproteobacteria formed the second largest class of Proteobacteria in all samples and consisted of Rhodobacterales, Rhizobiales, and an unclassified order at low salinity whereas at high salinity Rhodobacterales and Rickettsiales dominated. While dominating the alphaproteobacterial contribution in the lowest (G) and highest (W) salinity brines, Rhodobacterales were absent from the medium high salinity brine (Supplementary Table S1, sheet 4). Among the Bacteroidetes, the classes Flavobacteria and Cytophaga dominated at low salinity whereas Sphingobacteria dominated in the two higher salinity ponds. Notably, these high salinity Sphingobacteria consisted for nearly 99% of the known halophilic bacterium *Salinibacter ruber* (Supplementary Table S1, sheet 5). Within the Firmicutes, Clostridia and to a lesser extent Bacilli were the dominant groups (Supplementary Table S1, sheet 6). Overall, at the genus level, the top three dominant genera amongst the bacteria that could be assigned at the genus level were *Pseudoalteromonas*, *Shewanella*, and *Clostridium* (Table 4). Furthermore, an unassigned bacterial genus and an unassigned proteobacterial genus were abundantly present.

**Table 4:** Dominant genera at the three sites.

Green	%	Red	%	White	%
Unclass. bacterial genus	12.2	*Halorubrum*	36.0	*Halorubrum*	22.2
*Pseudoalteromonas*	8.7	*Natronomonas*	5.2	*Haloferax*	11.5
*Shewanella*	7.8	*Haloferax*	5.2	*Natronorubrum*	5.6
Unclass. Proteobact. genus	4.7	Unclass. Bacterial genus	4.3	*Natronomonas*	4.1
*Clostridium*	4.0	*Halobacterium*	2.3	*Natronolimnobius*	3.4
*Vibrio*	3.7	*Halomonas*	2.3	Unclass. bacterial genus	3.2
*Fusibacter*	2.6	Unclass. Gammaprot. genus	2.0	*Halobacterium*	2.8
*Pseudomonas*	2.4	*Haloarcula*	1.9	*Haloarcula*	2.2
Unclass. Alphaprot. genus	1.7	*Pseudomonas*	1.8	*Haloquadratum*	2.1
*Halomonas*	1.3	*Natronorubrum*	1.8	*Halosimplex*	1.9
*Photobacterium*	1.3	*Salinibacter*	1.7	*Haloterrigena*	1.5

More than 97% of the archaeal reads belong to the class of Halobacteria, while only a few methanobacterial reads were found. In fact, at the two highest salinities, Halobacteria made up approximately 63% of all rRNA reads in contrast to a mere 6% at the lowest salinity. The halobacterial reads were distributed over three families, the Haloferacaceae at 50–70% relative abundance with *Halorubrum* and *Haloferax* as dominant genera, Halobacteriaceae at 23–30% with *Natronomonas* as dominant genus at high salinity and *Haloarcula* at the lowest salinity, and Natrialbaceae at 6–19% relative abundance with *Natronorubrum* and *Natronolimnobius* as dominant genera at the highest salinities and *Natronococus* at the lowest salinity (Supplementary Table S1, sheet 7). *Halorubrum*, *Haloferax*, and *Natronomonas* are also overall the three most abundant prokaryote genera in the two hypersaline brines (Table 4). Most of the micro-eukaryote 18S rRNA sequences in the metagenomes belong to the Chlorophyta family of Dunaliellaceae with 1,057, 1,368, and 1,602 reads for sites G, R, and W, respectively. Most of these reads (70–94%) were assigned to *Dunaliella salina* (Fig. 3A).

Diversity indexes from the metagenome dataset revealed the highest number (4332) of observed species (Chao-1) at the lowest salinity site (G), which also had the highest evenness (0.04) and Shannon diversity index (4.8). For the two high salinity sites identical Shannon diversity (3.1) and evenness (0.01) were calculated while the observed species richness indexes were also similar (2682 and 2686 for R and W, respectively).

**Figure 4:**
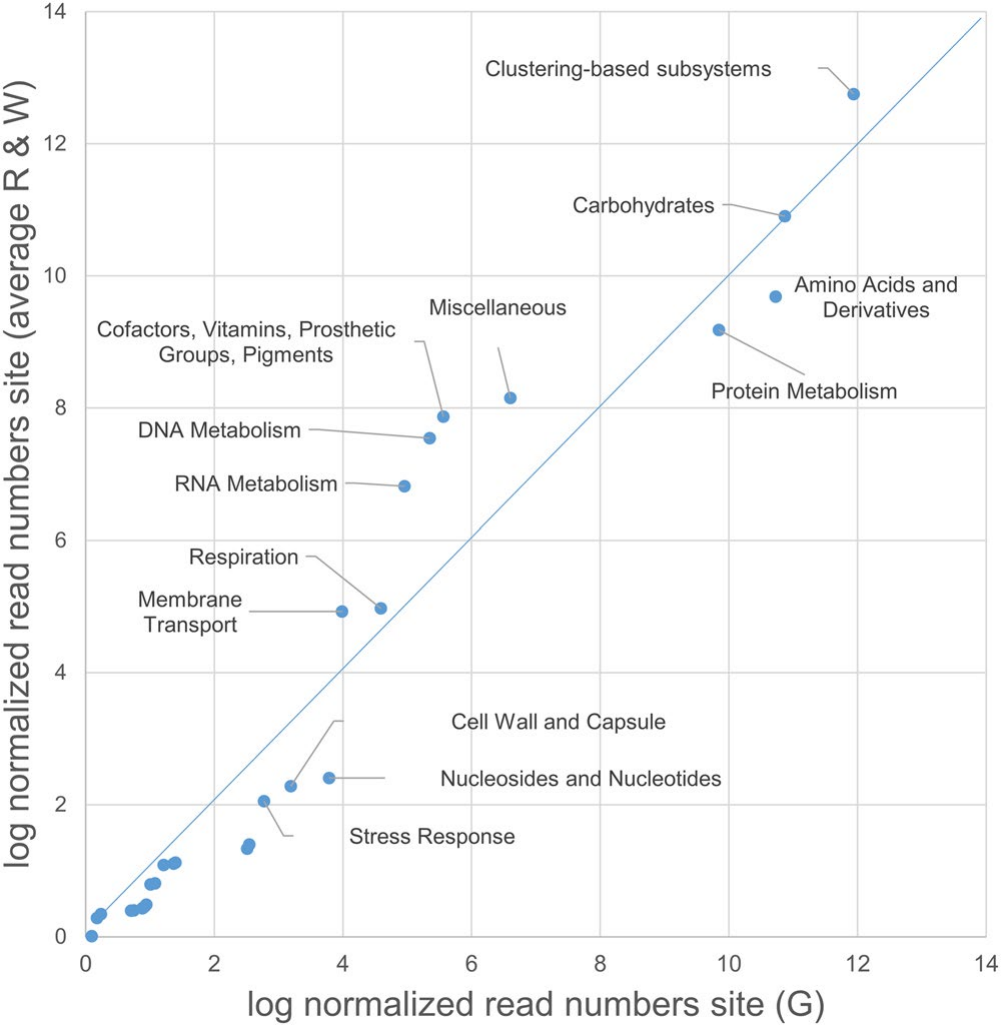
Relative gene ontology distribution between the lower (G) and higher (average of W and R) salinity sites. The blue line marks the 1:1 ratio line leaving Gene Ontology (GO) groups above the line as more pronounced at high salinity and GO groups below the line are more pronounced at lower salinity.

From the metagenome we extracted data of functions as defined by the gene onthology set (GO-slim)^19^. The distribution of functions was highly similar between the red and white brine and differed only slightly from the green brines. Comparison of the low salinity brine with the average of the high salinity brines revealed only small differences in functional diversity (Fig. 4). Cofactors-vitamins-prosthetic groups-pigments, DNA metabolism, and RNA metabolism are functions that occurred in higher numbers in the hypersaline sample, whereas processes related to nucleosides and nucleotides, cell wall and capsule, stress response, fatty acids-lipids-isoprenoids, and virulence-disease-defense were more pronounced in the low salinity brine. A list of most abundant genes was generated from the MG-RAST subsystems dataset (Table 5). In total, 345 protein-coding genes were identified at 55% amino acid identity (data not shown). The top ten most abundant genes made up 43.7% of the reads from the green brine, 69.7% of the red brine and 81.5% of the white brine. Table 5 revealed no overlap in dominant functions between the three brines.

**Table 5:** Abundantly identified proteins in the MG-RAST subcategories dataset at the function level.

Green	%	Red	%	White	%
Excinuclease ABC subunit A	9,7	Cell division transporter, FtsE (TC 3.A.5.1.1)	18,3	Flagellin FlaB	41,3
ATP synthase beta chain (EC 3.6.3.14)	7,1	NADH-ubiquinone oxidoreductase (EC 1.6.5.3)	11,0	Glycerol kinase (EC 2.7.1.30)	11,4
Iron-sulfur cluster assembly protein SufB	4,2	Ornithine carbamoyltransferase (EC 2.1.3.3)	6,9	Propionyl-CoA carboxylase beta chain (EC 6.4.1.3)	6,9
RecA protein	3,9	SSU ribosomal protein S23e (S12p)	6,3	DNA mismatch repair protein MutS	3,9
Excinuclease ABC subunit B	3,8	Lipid A export MsbA (EC 3.6.3.25)	5,7	3-hydroxyacyl-CoA dehydrogenase (EC 1.1.1.35)	3,5
S-adenosylmethionine synthetase (EC 2.5.1.6)	3,5	Deacetylase	5,1	Chaperone protein DnaK	3,0
Citrate synthase (si) (EC 2.3.3.1)	3,2	Cytidine deaminase (EC 3.5.4.5)	4,8	Cobyric acid synthase	3,0
Prolyl-tRNA synthetase (EC 6.1.1.15)	3,0	Glycine cleavage system H protein	4,3	Aldehyde dehydrogenase (EC 1.2.1.3)	2,8
Copper-translocating P-type ATPase (EC 3.6.3.4)	2,6	Putrescine transport PotA (TC 3.A.1.11.1)	3,8	Phosphonate ABC transporter (TC 3.A.1.9.1)	2,8
2,3,4,5-tetrahydropyridine-2,6-dicarboxylate N-succinyltransferase (EC 2.3.1.117)	2,5	Glutamyl-tRNA(Gln) amidotransferase (EC 6.3.5.7)	3,6	Homoisocitrate dehydrogenase (EC 1.1.1.87)	2,6
Total	43,7	total	69,7	total	81,5

**Figure 5:**
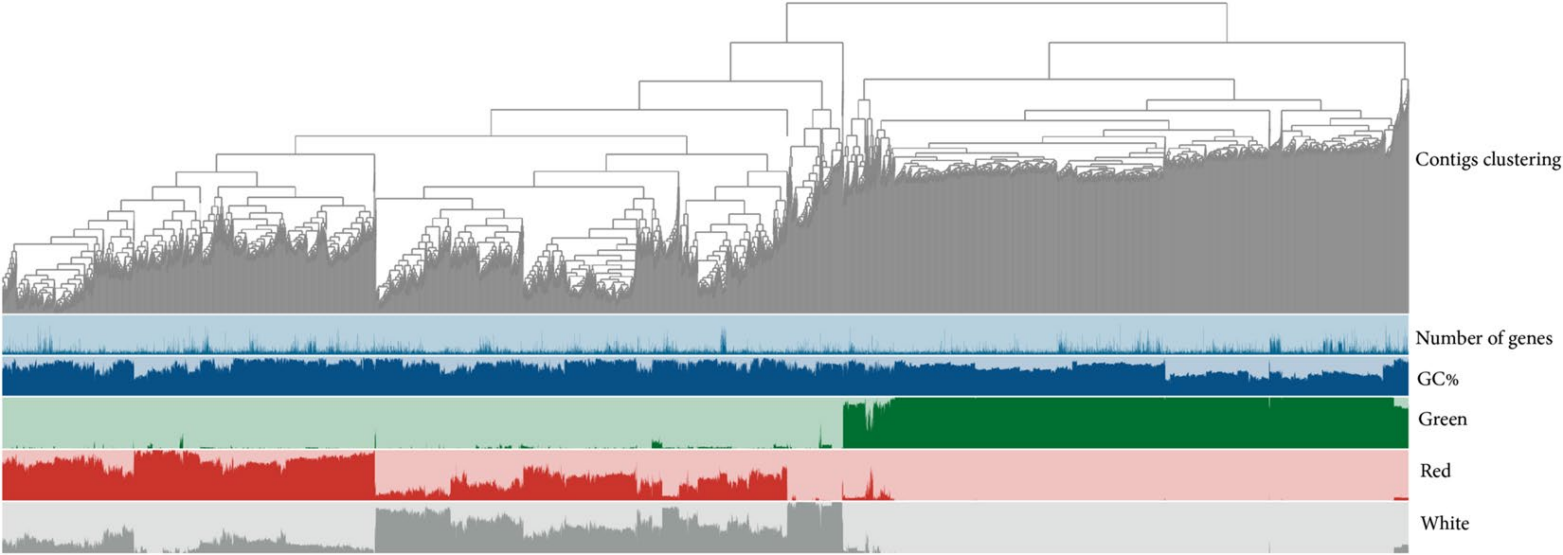
Common and sample specific sequences as revealed by metagenomic analysis. Hierarchical clustering of contigs based on their sequences and sample coverage. The dendrogram tips represent the splits of contigs (produced by Anvi’o) so that the maximum length of the contig did not exceed 20,000 bp. The number of genes and GC% refer to the number of ORFs identified in a given contig split and the respective GC content of these contigs. The size of each bar in the graphs marked Green, Red and White represents the relative abundance of each read that aligns with a given contig split of a given sample.

The co-assembled reads database of the metagenome contained 25,406 contigs with a minimum contig length of 2,500 bp, which corresponded to 27% of all contigs and 59% of all nucleotides found in the database. The contigs were organized in 60 bins with 2 to 2,609 contigs per bin. The contigs in the bins were split to ensure that no read exceeded 20,000 bp. The total number of splits (corresponding to tree tips of the dendrogram, representing the shared and unique bins) was 25.749, which are plotted on the X-axis (Fig. 5). The GC content varied from 34.1 to 70.5%. Applying a cut-off of 1% recruitment (of the total number of reads from a sample site), only 1 bin (number 20) contained (split) contigs that aligned with reads from all three sample sites. However, 11 (bin numbers 1, 2, 3, 7, 11, 13, 14, 20, 25, 26, and 28) were shared between the red and white sample sites at a recruitment of >1%. When considering a mean coverage higher than 1% (the number of reads of a sample that aligns with a contig in a particular bin), only three bins (number 20, 34, and 48) contained contigs that aligned with reads from all three sites. Figure 5 shows that the green site was very different from the two higher salinity sites and that there was hardly overlap. The red and white sites also form distinct clusters but there was more overlap between them. Moreover, it is remarkable that the low salinity green site was characterized by a lower GC content (average 54%) compared to the high salinity sites (average 61%).

For the comparison of genera obtained by isolation with genera identified by metagenomics analysis (MG-RAST and read assembly), samples from the different stations were combined (Tables 6 and S1 sheet 11). The bacterial genera *Halomonas* and *Pseudomonas* are abundant amongst all datasets (top 10). *Idiomarina* are abundant amongst the isolates and contigs but rank lower in the MG-RAST dataset. Several isolates, e.g. *Bacillus*, *Marinobacter* and *Streptomyces*, are present in the MG-RAST dataset but are not found the annotated contigs. On the other hand, isolates belonging to the genus *Algoriphagus*, *Kocuria* and *Spiribacter* are present in the assembled contigs but absent in the MG-RAST annotation. Interestingly, nine of isolated genera were not found in the two metagenome datasets. *Salinibacter*, *Clostridum* and *Shewanella* are among the major genera present in the metagenome datasets but absent from the isolates. For the Archaea, only seven genera were isolated (Tables 6 and S1 sheet 11) and only three genera were identified by their rRNA in the metagenome assemblies while thirty-four were annotated by MG-RAST (Supplementary Table S1 sheet 11). *Haloarcula* was the only genus present in all three datasets and identified at high numbers. *Halorubrum* was the dominant genus among the isolation and MG-RAST annotation but is absent from the assembly. Abundant genera like *Haloferax*, *Natronomonas*, *Natronorubrum* and *Halobacterium* are only found in the MG-RAST annotated dataset (Supplementary Table S1 sheet 11).

**Table 6:** Comparison of major genera in the combined samples identified by cultivation with genera found in the MG-RAST annotation and annotation of rRNA containing assembled contigs.

	Isolates^1^	MG-RAST-rRNA^1^	Contigs-rRNA
**Bacteria**			
*Halomonas*	9	1272 (4)^2^	26 (1)
*Idiomarina*	7	135 (43)	18 (2)
*Algoriphagus*	4	—^3^	4 (19)
*Bacillus*	4	195 (29)	—
*Marinobacter*	4	322 (14)	—
*Salimicrobium*	3	—	—
*Aliidiomarina*	2	—	—
*Pseudomonas*	2	1313 (2)	9 (9)
*Streptomyces*	2	519 (7)	—
*Thalassobacillus*	2	—	—
*Actinopolyspora*	1	—	—
*Albirhodobacter*	1	—	—
*Alcanivorax*	1	52 (87)	1 (32)
*Ancylobacter*	1	4 (279)	—
*Arhodomonas*	1	16 (182)	—
*Gracilibacillus*	1	—	—
*Kocuria*	1	—	1 (46)
*Leifsonia*	1	81 (63)	—
*Loktanella*	1	24 (151)	5 (15)
*Oceanobacillus*	1	—	—
*Paracoccus*	1	54 (84)	—
*Piscibacillus*	1	—	—
*Pseudoalteromonas*	1	335 (13)	14 (4)
*Salicola*	1	—	—
*Salinivibrio*	1	71 (71)	—
*Spiribacter*	1	—	6 (13)
*Vibrio*	1	294 (17)	9 (11)
*Virgibacillus*	1	34 (112)	—
**Archaea**			
*Halorubrum*	16	22143 (1)	—
*Natrinema*	14	35 (30)	—
*Haloarcula*	11	2648 (4)	1829 (1)
*Haloterrigena*	4	627 (17)	—
*Halovarius*	1	—	—
*Halonotius*	1	—	—
*Halovivax*	1	158 (25)	—

^1^To allow comparison with the metagenomic assembly dataset that was obtained by pooling sequence reads from all samples, the sum of isolates and MG-RAST annotations from the three samples are presented.

^2^Between brackets is their relative position according to decreasing abundance. Note that several genera are found at identical numbers and are positioned according to rank and alphabet.

^3^The minus sign indicates that the genus is not identified in the dataset.

## Discussion

Based on its inland location, unconnected to the sea and the input of mainly riverine freshwater enriched with salts leaching from its immediate environment, an athalassohaline composition was expected. Lake Meyghan has a thalassohaline composition, which is not uncommon for inland hypersaline lakes^11, 12, 16, 20, 21^. However, molecular and cultivation analysis revealed organisms that are commonly found in thalassohaline and athalassohaline lakes (see below). The total number of 16S rRNA gene copies in the genomes of each community member is unknown and reports suggest copy numbers in fully sequenced bacterial genomes ranging from 1 to 15 genes per genome with an average of 4^22^. Total cell counts in the hypersaline basins estimated from gene copy numbers are in the range of 10^6^ cells mL^−1^, similar as reported for other hypersaline environments worldwide^23–26^, but lower than the microbial cell densities of 10^8^ or more that were reported by others^27, 28^. The number of identified sequence reads is relatively low as compared to the initial 30 million reads per sample that passed quality control but is a consequence of still incomplete reference databases, especially when less common ecosystems like hypersaline lakes are analyzed.

The green site had salinities similar to - for example - the Salton Sea, USA, which has a salinity of about 56 g kg^−1^ ^29, 30^. The metagenome of the green - low salinity - site contained several chloroplast sequences, sequences from the eukaryal phyla Bacillariophyta (diatoms, mainly of the genus *Phaeodactylum*) and Chlorophyta (mainly of the genus *Dunaliella*), as well as a low number of Cyanobacteria-related sequences. Both a diverse phytoplankton community and low Cyanobacterial abundance were also found in the Salton Sea^31^, arguing that the composition of the green site is typical for basins with a slightly elevated salinity relative to seawater. The abundantly present chlorophyll-containing microbes may explain the color of the green site. These organisms represent the primary producers in this ecosystem, fixing carbon and producing organic matter in the form of sugars. The majority of the green site’s microbial diversity consists of Bacteria, typical for low salinity ecosystems and mostly consisting of heterotrophic species that decompose the primary produced carbon sources. Despite the large distance from the nearest sea, the bacterial diversity was typical for marine ecosystems or for systems with an elevated salinity^29, 30, 32^. For example, the dominantly occurring bacterial genera *Pseudoalteromonas*, *Shewanella*, and *Vibrio* are commonly found in ocean waters^33–35^. Not common to normal marine waters, however, is the occurrence of haloarchaea such as *Haloarcula*, *Haloferax*, *Natronococcus*, *Halogeometricum*, and even the extreme halophilic *Haloquadratum*. Two halophilic genera were even more abundant in the green basin than in the hypersaline basins: *Haloarcula* and *Natronococcus*. Halophilic species such as *Haloquadratum* should not survive at a salinity of 50 g kg^−1^. Moreover, presence of haloarchaea has not been documented in any of the Salton Sea studies^29, 30, 32^. This raises the question whether members of these species are capable of (temporarily) resisting these low salinities or whether their sequences were derived from an extracellular pool of DNA that might have been co-extracted from the samples. Given the absolute requirement for high salt concentrations of species such as *Haloquadratum*, the extracellular pool of DNA seems the best explanation for the detection of sequences belonging to these organisms in the metagenome. However, the isolation of at least 4 halophilic Archaea from the green basin using hypersaline isolation media suggests that some of these species resisted the low salt concentrations. Indeed, species such as *Natronococcus* are capable of growing at low salinities^36^. An experiment where samples from marine wetlands (32 g kg^−1^ salinity) were inoculated on high salinity media (100–200 g kg^−1^) yielded several haloarchaeal isolates related to *Halorubrum*, *Haloferax*, and members of the family of Natrialbaceae^37^. While this demonstrates that these isolates resist low salinities, they require higher salinities for optimal growth, in accordance with the 4 archaeal isolates presented here, which were isolated on 230 g kg^−1^ salinity media. A potential source of these sequences and strains could be the occasional discharge of hypersaline water into the lower salinity areas of Lake Meyghan resulting from the industrial sulfate mining.

As expected, the microbial composition in the green - low salinity - basin differed greatly from those of the two hypersaline basins, which were dominated by haloarchaea. The red basin might derive its color from alga like *Dunaliella salina* and from halophilic archaea. Although *Dunaliella* belongs to the family of green algae, they often have a red appearance at high salinity due to the high content of carotenoids^38^. Haloarchaea are also rich in carotenoid derivatives such as bacterioruberin that protects the organism against oxidative DNA damaging agents such as UV irradiation^39^. Although the same organisms were present in the white basin, their characteristic red color was probably masked by the high precipitation of salts. Especially at high salinities, *Dunaliella salina* becomes the last surviving primary producer that feeds the heterotrophic community mainly through the production and release of the osmoprotectant glycerol^1, 10^.

The slightly alkaline nature of Lake Meyghan (pH 7.7–8.8) raised the question whether the brines are home to haloalkaliphiles, species that require both high salinity and a high pH for optimal growth^40^. However, the highest pH of 8.8 was restricted to the low salinity green basin while the hypersaline brines with pH < 8 were below the threshold of >pH 9 for alkaline waters^40^. The halophilic members found in the red, medium salinity basin consisted mainly of genera commonly found in thalassohaline brines such as the haloarchaea *Halorubrum*, *Haloferax*, *Halobacterium*, and *Haloarcula* and halophilic bacteria such as *Halomonas* and *Salinibacter*. However, genera such as *Halorubrum*, are also commonly found in haloalkaline environments^41^. In the extreme hypersaline, white basin, however, several genera that were more abundant are common to athalassohaline high pH brines e.g. *Natronorubrum*, *Natronomonas*, and *Natronolimnobius*. Also among the isolates are species typically associated with alkaline hypersaline waters such as *Natrinema* sp.^41^, of which 9 strains were isolated from the red brine and 4 strains from the white brine. However, the same brine also contained members of the genus *Haloquadratum*, genera typically found at neutral to slightly basic pH in saturated crystallizer ponds^25^. Moreover, strains of the haloalkaliphile *Natronomonas* were also found at lower pH while other characteristic genera for haloalkaline ecosystems such as *Natrialba* and *Thioalkalivibrio* ^40^ were present at low numbers. Despite the large overlap in community composition between the red and white brines, there were some noticeable differences mostly at the lower taxonomic levels. Notably, the SSU reads for the bacterial phyla Planctomycetes and Cyanobacteria were present in the green and white basins but were not found in the medium salinity red basin. Also, SSU annotated reads from the alphaproteobacterial order Rhodobacterales were not found in the red brine while in contrast the Rhizobiales were nearly absent from the white brine. Amongst the gammaproteobacterial orders, Oceanospirillales and Xanthomonadales were more abundant in the red brine while Enterobacteriales and Legionellales were more abundant in the white brine. Two gammaproteobacterial strains that were isolated from the white brine are potential novel species having a sequence identity less than 96% with known species. One isolate has 95.2% identity with *Arhodomonas recens* RS91, a halophilic alkane-utilizing hydrogen-oxidizing bacterium^42^ and the other has 95.7% identity with *Spiribacter salinus* M19–40, an abundant halophilic bacterium isolated from 190 g kg^−1^ ponds of salterns on Isla Christina, Spain^43^. Among the haloarchaea, genus *Halalkalicoccus*, *Halobiforma* had a higher relative abundance in the red brine while genera like *Haloquadratum* were more abundant in the high salinity brine. The preferred prevalence of strains at either highest or medium salinity most likely reflected their competitive advantages at these salinities. For example, it is well established that *Haloquadratum* dominates at the highest salinities by efficiently competing for the extant resources and by being able to resist the imposed desiccation stress^27^.

Binning of the assembled reads confirmed the overlap in diversity between the two higher salinity sites and their difference with the green site. Only a small overlap in sequences was found, potentially related to the previously mentioned haloarchaea in the green basin. Also, the observed difference in GC content was typical for hypersaline ecosystems where the majority of haloarchaeal species had a high GC content whereas at lower salinities the lower GC Bacteria dominated^44^. An exception is *Haloquadratum walsbyi*, which led to a significantly different GC signature in crystallizer ponds of the Spanish, Santa Pola solar salterns, in agreement with the abundance of this organism in the crystallizers. In Lake Meyghan, however, the contribution of *Haloquadratum* barely exceeded 2% and therefore did not influence the overall GC content.

Despite significant differences in the community composition, the functional properties of the different brines as deduced from the COG distribution did not greatly differ. Most likely, this reflected the conserved ecosystem functioning of saline lakes and brines that largely consist of photosynthetic primary producers and heterotrophic decomposers while higher organisms and predators are absent or limited to the smaller brine shrimps, *Artemia*. Similar conservation in ecosystem function was found in microbial mats ranging from hypersaline, hot spring, to coastal microbial mats^45^. Only at the gene-level, variations between the brines were found, including a decrease in number of assigned functions in both hypersaline brines. This probably reflected the lower diversity of the two hypersaline brines with less species being able to thrive under these conditions. In the most saline, white brine, flagella synthesis appeared important, which may aid the cells in chemo- and phototaxis. The abundantly present glycerol kinase genes is in agreement with glycerol as major carbon and energy source derived from algae like *Dunaliella* sp^38^. However, metagenome analysis cannot predict gene expression and protein synthesis and for a thorough analysis of functional complexity in microbial ecosystems, a metatranscriptomic or proteomic approach is essential.

In general, when isolating microorganisms, the results often differ from the actual distribution of microbial taxa in that environment due to the inability to culture a large proportion of microbial species while opportunistic rare species dominate the isolates^46^. The most abundant bacterial genera isolated, *Halomonas* and *Idiomarina*, and to a lesser extent *Pseudomonas* and *Streptomyces*, are also abundant in the metagenome datasets suggesting that the isolates give a reasonable representation of the dominant diversity. A similar result was obtained for the halobacterial genera of *Halorubrum* and *Haloarcula*. However, many more genera are identified by metagenomics and several of these may not be isolated in the near future due to specific yet unknown growth requirements. Isolation yielded several species that probably belong to the rare biodiversity that are not sufficiently present to be detected by metagenomics but may be opportunists that easily grow under the applied cultivation conditions. A better comparison between cultivation and metagenomics would require a multitude in isolates grown under variable conditions.

## Methods and Materials

### Sampling sites and sample collection

Lake Meyghan (34°11′–27.91″N, 49°50′–26.70″E) was sampled in November 2013 at the peak of the dry season. The shallow brines were sampled at three different sites that were named according to the brine color. These were G (green, 34°11′–21.59″N, 49°50′–45.73″E), R (red, 34°11′–20.78″N, 49°50′–21.87″E), and W (white, 34°11′–35.31″N, 49°50′–18.25″E) (Fig. 1). Thirty liter samples were taken aseptically and transferred to sterile plastic containers and were brought to the laboratory within a few hours. Total DNA was extracted from ~20 liters of sample that was prefiltered over 20 μm (ALBET, Germany) filters and subsequently filtered over multiple 0.2 μm (Sartorius, Germany) nitrocellulose filters until they clogged. The remainder of the samples was stored at 4 °C for physicochemical analyses and culturing. Aliquots of the samples were sent to a commercial water chemistry laboratory (Khak Behin Azma Co., Iran) for analysis of the chemical composition.

### Culture media and growth conditions

Bacteria and Archaea were isolated from the samples under aerobic conditions on four different growth media. Neutral Oligotrophic Medium^47^ (NOM, 240 g kg^−1^ salinity) for haloarchaea consisted of (in g L^−1^): NaCl 184.0, MgSO_4_.7H_2_O 26.8, MgCl_2_.6H_2_O 23.0, KCl 5.4, sodium pyruvate 1.0, K_2_HPO_4_ 0.3, CaCl_2_.2H_2_O 0.25, NH_4_Cl 0.25, fish peptone 0.25, yeast extract 0.05, and agar 20.0 with a pH of 7.3. Modified Growth Medium (http://www.haloarchaea.com/resources/halohandbook) (MGM, 230 g kg^−1^ salinity) contained (in g L^−1^): NaCl 184.8, MgSO_4_.7H_2_O 26.9, MgCl_2_.6H_2_O 23.1, peptone 10.0, KCl 5.4, yeast extract 2.0, CaCl_2_.2H_2_O 0.8, and agar 15.0 with a pH of 7.2. The Moderately Halophilic Medium^48^ (MHM, 100 g kg^−1^ salinity) contained (in g L^−1^): NaCl 81.0, MgSO_4_.7H_2_O 9.6, MgCl_2_.6H_2_O 7.0, KCl 2.0, CaCl_2_ 0.54, glucose 1.0, proteose peptone 5.0, yeast extract 10.0, and agar 15.0, NaBr 0.026, and 10.0 mL NaHCO_3_ solution (0.06 g NaHCO_3_ in 10.0 mL deionized water); pH was set a 7.5. Marine Medium^49^ (MM, 30 g kg^−1^ salinity) contained (in g L^−1^): NaCl 19.45, MgCl_2_ (anhydrous) 5.90, peptone 5.0, Na_2_SO_4_ 3.24, CaCl_2_ 1.80, yeast extract 1.0, KCl 0.55, NaHCO_3_ 0.16, Fe (III) citrate 0.10, KBr 0.08, SrCl_2_ 0.034, H_3_BO_3_ 0.022, Na_2_HPO_4_ 0.008, Na_2_SiO_3_ 0.004, NaF 0.0024, (NH_4_)NO_3_ 0.0016, and agar 15.0 with the pH set at 7.6. All samples were serially diluted up to 10^−6^ and plated according to Burns *et al*.^25^. The plates were incubated aerobically for 8 weeks at two different temperatures: 30 °C and 40 °C. Isolated colonies of microorganisms were selected according to their size, shape, and color and streaked on new agar plates with the same growth medium. This procedure was repeated at least three times until pure cultures were obtained.

### Nucleic acid extraction, amplification and sequence analysis

Genomic DNA was extracted from colonies grown on the agar medium from which the strain was isolated, using the Genomic-DNA extraction kit (Roche, Diagnostic, Mannheim, Germany) and according to the manufacturer’s recommendations. The DNA concentration and purity were assayed using the Nanodrop 1000 spectrophotometer (Thermo Scientific, Wilmington, DE, USA) and were confirmed by visualization on a 1% agarose gel. The 16S rRNA genes from the isolates were amplified using either the Bacteria specific primers 27F and 1492R or the Archaea specific primers 20F and 1530R (Table 7). The PCR conditions were as follows for Bacteria: 94 °C for 3 min, followed by 25 cycles of 95 °C for 45S, 55 °C for 45S and 72 °C for 90S and a final 10 min extension at 72 °C and for Archaea: 94 °C for 3 min, followed by 30 cycles of 94 °C for 15S, 52 °C for 30S and 72 °C for 50S and a final 7 min extension at 72 °C. Sanger sequencing was performed on an ABI 3730XL DNA sequencer at Macrogen (Seoul, South Korea) generating on average 900 bp sequences using bacterial 27F or the archaeal 20F oligonucleotides as sequencing primers. Neighboring taxa were identified using the BLASTN program and analysed by pairwise sequence alignment to calculate sequence similarity using the EzBioCloud server (www.ezbiocloud.net)^50^. The sequences were considered to belong to an operational taxonomic unit (OTU) when sharing ≥97% sequence identity.

**Table 7:** List of oligonucleotides used in this study.

Primer Name	Primer Sequence (5′ to 3′)	Reference
27F	AGAGTTTGATCMTGGCTCAG	Lane^69^
1492R	GGTTACCTTGTTACGACTT	Lane^69^
20F	TCCGGTTGATCCTGCCG	Xin *et al*.^70^
1530R	AAGGAGGTGATCCAGCC	Lane^69^
Eub338	ACTCCTACGGGAGGCAGCAG	Amann *et al*.^71^
Eub518	ATTACCGCGGCTGCTGG	Muyzer *et al*.^72^
Parch519F	CAGCCGCCGCGGTAA	Ovreås *et al*.^73^
ARC915R	GTGCTCCCCCGCCAATTCCT	Ovreås *et al*.^73^

For metagenomic analysis, multiple filters were pooled for DNA extraction using a phenol-chloroform based protocol^51^. Metagenome sequencing was performed using a paired-end protocol with an average read size of 100 bp (PE100) on Illumina HiSeq 4000 (BGI, Hong Kong) on randomly sheared 170–500 bp DNA fragments.

### Bioinformatic data analysis

Taxonomic and functional profiling of raw sequencing reads was performed on the MG-RAST pipeline that contains its own quality control algorhythm^52^. Taxonomic profiles of the microbial community were extracted from the MG-RAST annotation pipeline focusing on sequences annotated as small subunit ribosomal (SSU) RNA using the Silva SSU database as a reference. For further in house analysis, low quality reads were removed using Trimmomatic^53^. The filtering cutoffs were: minimum length 100 bp, average quality score 20, sliding window 4:20, maximum info 100:0.8.

Metagenomic contigs were annotated using a collection of *in house* bioinformatics scripts (supplementary material S2). The pipeline included: the identification of ORFs with Prodigal^54^, tRNAs with tRNAscan-SE^55^ and functional annotation using BLAST^56^ implemented in USEARCH^57^ and HMMER-3^58^. The databases used for taxonomical and functional annotation were: Silva SSU release 123^59^, COG^60^, TIGRFAM^61^ and NCBI non-redundant RefSeq release 75^62^. Comparative metagenomic analysis of the three samples was applied in order to overcome the shallow sequencing of the metagenome and to identify sequences that appear in all samples and those that are sample specific. Quality filtered reads were co-assembled using MEGAHIT^63^ with a minimum contig length of 1,000 bp. Reads from each metagenome were mapped back to the contigs using bowtie-2^64^. Anvi’o pipeline^65^ metagenomics workflow was applied to build a contigs database and to analyze the distribution of functional annotation of open reading frames and GC% across the individual samples. Briefly, reads of each sample were mapped to the co-assembled contigs and bins of clustered contigs were generated and stored in a phylogenetic tree applying the default settings of CONCOCT^66^. Hierarchical clustering was done by combining the sequence alignments with the differential coverage of relative abundance of individual sample reads. The individual sample reads were aligned to the sequences in the contig bins using Euclidean distance with a maximum contig length of 20,000 bp.

### Quantitative real time PCR (qPCR)

The abundance of 16S rRNA genes (bacterial and archaeal) in each sample was measured using qPCR. The standard curves and qPCR were performed as described by Nathani *et al*.^67^. Briefly, plasmid DNA possessing a full-length copy of the 16S rRNA gene belonging either to the *Halorubrum chaoviator* (DSM 19316) or *Escherichia coli* (ATCC 25922) were used as DNA standards in qPCR. The target DNA for standard curves was amplified using the domain-specific primer sets 27F and 1492R for Bacteria and 20F and 1530R for Archaea. Copy number per µl of extracted DNA was calculated using formula: Copy number per µl = (concentration of plasmids (gm/µl) × 6.023 × 10^23^)/(length of recombinant plasmid (bp) × 660), where 660 is the molecular weight of one base pair in double-stranded DNA and 6.023 × 10^23^ = Avogadro’s number^68^. qPCR was performed with Rotor-Gene^TM^ 6000 (Corbett Research Biosciences, Sydney, Australia) using the domain-specific primer sets Eub338 and Eub518 to detect Bacteria and Parch519F and ARC915R to detect Archaea (Table 7). Amplifications were performed in a total volume of 15 μl, containing 30 ng of template DNA, 7.5 μl of 2× Maxima SYBR Green qPCR Master Mix (Fermentas, France), 1.5 μl of each primer (10 pmol/μl) and sterile H_2_O to a total volume 15 µl. The amplification program involved an initial denaturation step at 95 °C for 15 min, followed by 40 cycles of 94 °C for 30S, 60 °C for 30S and 72 °C for 20S for both Bacteria and Archaea. For all standard curves, the coefficients of determination (R^2^ value) were better than 99.0%. The number of target genes per ml of sample was calculated using formula:

Number of gene copies per mL samples = (gene copies per reaction mixture × volume of DNA (µl))/(3 µl DNA per reaction mix × volume of sample (ml))^62^.

### Nucleotide sequence accession numbers

The 16S rRNA gene sequences determined for the isolates in this study have been deposited in GenBank with accession numbers KY411714-KY411818. The metagenomic data is publically available at MG-RAST under the accession numbers: 4683415.3, 4683416.3 and 4683417.3 and at the EBI metagenomics server under accession numbers ERS1455389, ERS1455390 and ERS1455391.

## Electronic supplementary material

Supplementary Dataset 1

Supplementary Dataset 2
