# Supplementary material for: Microbial diversity in the hypersaline Lake Meyghan, Iran
Source: Sci Rep. 2017 Sep 14;7:11522. doi: 10.1038/s41598-017-11585-3 (PMC5599592; doi:10.1038/s41598-017-11585-3)
Supplement: Supplementary file 2 — Supplementary Dataset 2 [file 41598_2017_11585_MOESM2_ESM.zip › Supplementary data S2.pdf]

```
#!/usr/bin/perl
#file annot_helper.pl

use strict;
use warnings;
use Data::Dumper;
use BIO::SeqIO;
use DBI;
use HTML::Table;

########
# cretoius@cshl/programmingforbiology2015
# contigs_annotation_helper
# scripts to help with the annotation of
metagenomic contigs via different programs
# the scripts worked for me and others are
free to use them, but I cannot guarantee any
usability
# in the event of errors my advise for you is
to check the manual of the softwares
(prodigal, usearch, tRNAscan-SE, hmmer3, BLAST
# or you can contact me at cretoiums@gmail.com
########
# https://www.cpan.org
# prodigal
# usearch
# hmmer3
# blast
# pfam
# tigr
# tRNA-Scan SE
# RefSeqnr (default)
########

annot_helper -input data.fna -output result

# Required input arguments
```

```
-input      fasta file
-ouput      prefix for output files

# Optional input arguments

-db             database to use for blast
(default RefSeq non-redundant v.75), path
required
-dbindex        database blast index, path
requiered
-intfiles       keep all intermediary files
-n              number of processors
-evalue         blast evalue

my $PIPELINE=“annotation”;
my ($input,$output,$help,$n,$evalue,$db,
$dbindex);

$evalue=0.00001;
$db=“myrefseq.udb”;
$dbxindex=“myrefseq.cdbfastaindex”;

my $seqin  = Bio::SeqIO->input(-file =>
"$input”, -format=>"fasta");
my $seqout = Bio::SeqIO->out(-file =>">
$inputindexed”, -format=>"fasta");

      my $numseqs;
      while (my $inseq = $seqin->next_seq) {
            my $id   = $inseq->display_id;

            if ($id =~ /_/) {
                  print "The input file name
cannot have identifiers containing an
underscore \n";
```

```
}
            $id=~s/\s+//g;
            $inseq->display_id($id);
            $seqout->write_seq($inseq);
            $numseqs++;

      }
      print "Checked $numseqs sequences. All
sequences are OK and annotation goes on \n";
      return $inputindexed;

}


# predict the genes with prodigal

my $prodigal_to_run = "prodigal2.pl -infile
$infile -meta -p $output $keep”;


# an output file ouput1.gbk is generated

my $gbk_finalfile="$ouput1.gbk";

# USEARCH

my $usearchinput=“output1.gbk”


usearch -ublast $ouput1.cds -db $db -maxhits 1
-evalue 0.00001  -userout $ouput1_CDS.usearch
-userfields query+target+id+alnlen+mism+opens
+qlo+qhi+evalue+bits+ql+tl+qs+ts+qcov+tcov
+pctpv";

# Annotation of pfam domains
```

```
my $dbh = DBI->connect('DBI:mysql' ,
$dbindex::dbname, $db);
my $sql = "select gp.gene_name,
gp.protein_length, d.domain_accession,
d.domain_name, dm.aln_start, dm.aln_end,
dm.e_value, d.domain_length,
ddb.domain_db_name, ddb.domain_db_version
           from gene_prediction gp,
domain_match dm, domain d, domain_db ddb,
contig c
           where gp.contig_id = c.contig_id
and gp.gene_prediction_id =
dm.gene_prediction_id and dm.domain_id =
d.domain_id and d.domain_db_id =
ddb.domain_db_id
             and c.assembly_id = $aid and
gp.gene_name = '$gid' order by dm.e_value";
$sth = $dbh->prepare($sql);
$sth->execute;

# move to the tRNAscan-SE

if ($flags{"tRNAscan"} == 1) {
   msg("Predict tRNAs with tRNAscan-SE",1);
    do_tRNAscan($thefile,
    $prodout,
    $db_config{“gccodefile”},
      $thekey);
    } else {
    print “Skip prediction of tRNAs \n;
    }

$output_files{$thekey}{"tRNAscan"} =
"$prodout.tRNAscan.out";

# and further concatenate the predicted ORFs
```

```
if ($flags{"concat_pep"} == 1) {
        print “Concatenate prodigal ORFs” \n;

my $pepfile = $output_files{$thekey}
{"prodigal.pep"};
        print “Further: cat $pepfile \>\>
$outfix.concat.pep" \n;

system ("cat $pepfile \>\>
$outfix.concat.pep");
    } else {
        print “Skip concatenate prodigal ORFs
\n;
    }
    $output_files{"concat.pep"} =
"$outfix.concat.pep";
}


# hmmer3
# move directly into hmmer3 with concat.pep
file and follow the default pipeline


# Blastp vs RefSeq non-redundant proteins
database

my $mydb=“refseqnr”
my $dbheader

if ($dbheader{“refseqns”} == 1) {

      print“Blastp is OK \ n);


do_parallel_blastp($output_files{"concat.pep.h
mmer.out”},
```

```
                        $db_config{“refseqnr”},
                        $outfix,

}
      else {
    print “Blastp error \ n” ;
}

      $output_files{“refseqout”} = "$outfix";
END

########
# No Errors
# resulting files are gff and can be easly
transformed in gbk or any format required for
downstream analysis
########
```
